# Editorial: Mitochondrial Dynamics in Endocrine Physiology and Disease

**DOI:** 10.3389/fendo.2022.844842

**Published:** 2022-02-16

**Authors:** Cecilia Poderoso, Beatrice Maria Filippi, Paula Mariana Maloberti, Maria Clara Franco

**Affiliations:** ^1^ Department of Human Biochemistry, Faculty of Medicine, University of Buenos Aires, Capital Federal, Ciudad Autónoma de Buenos Aires, Argentina; ^2^ Instituto de Investigaciones Biomédicas, Facultad de Medicina, Universidad de Buenos Aires, Ciudad Autónoma de Buenos Aires, Argentina; ^3^ School of Biomedical Sciences-Faculty of Biological Sciences-University of Leeds, Leeds, United Kingdom; ^4^ Department of Biochemistry and Biophysics, College of Science, Oregon State University, Corvallis, OR, United States

**Keywords:** mitochondrial fusion, mitochondrial fission, neurodegenerative diseases, mitochondrial metabolism, endocrine system, cellular metabolism

Mitochondria are important energy sensors; they modulate their activity to meet the energy demands of the cell. Changes in the cellular nutritional status are sensed by the mitochondria, eliciting a response that ranges from changes in mitochondrial morphology to alterations in the efficiency of oxidative phosphorylation. For example, when the cellular energy demand increases, mitochondria tend to fuse together (fusion) to increase the efficiency of oxidative phosphorylation and ATP production. Conversely, an excess of energy can lead to mitochondria separation (fission). These dynamic changes are tightly modulated and appear to be dysregulated in many metabolic and endocrine diseases. In this Research Topic, we aim to bring together complementary reviews, perspectives, and original research articles reflecting on how mitochondria dynamics regulate the normal physiological and endocrine functions, and how the alteration of these functions associates with disease processes.

A set of mitochondria-shaping proteins control mitochondria dynamics and determine whether fusion or fission will occur. Here, Yu et al. highlighted the most recent insights on the molecular mechanism associated with mitochondria dynamics. The authors particularly focus on emerging roles for the mammalian mitochondrial proteins mitochondrial fission protein 1 (Fis1), mitochondrial fission factor (Mff), and mitochondrial elongation factors 1 and 2 (MIEF1 and MIEF2) in regulating the recruitment of the cytosolic dynamin-related protein (Drp1) to the surface of mitochondria. They also address how these proteins, especially Fis1, mediate crosstalk between the mitochondrial fission and fusion machineries. Ihenacho et al. reviewed the involvement of Fis1 in the regulation of mitochondria fission, and its particular association with mitochondria-targeted for degradation by autophagy. In relation to the fission protein Dpr1, the original article by Schultz et al. explored the role of MiD51 in promoting fission by anchoring Drp1 to the mitochondrial membrane. Overexpression of MiD51 in human pancreatic cells caused mitochondrial fragmentation, while downregulation of MiD51 led to inhomogeneity of the mitochondrial network and hyper-elongated mitochondria. These alterations were associated with changes in membrane potential, oxygen consumption rate, and glucose-stimulated insulin secretion.

It is well established that mitochondria also participate in steroidogenesis. In particular, the steroidogenic acute regulatory protein (STARD1) plays a crucial role in transporting cholesterol from the outer to the inner mitochondrial membrane. In this Research Topic, Larsen et al. reviewed the involvement of STARD1 in the mitochondrial cholesterol metabolism looking at different STARD1 mRNA synthesis, splicing, and processing associated with distinct tissues and steroidogenic cell types. The role of mitochondria in sex hormone-induced energetic disorders was also discussed in this Research Topic. Yin et al. reviewed the effects of androgens and estrogens on mitochondrial energy metabolism in the regulation of insulin sensitivity. The authors proposed a hormone synergy-based view for understanding the complex effects of androgens in the control of insulin action in men with hypogonadism and women with polycystic ovary syndrome. Inherited metabolic disorders like glycogen storage disease subtypes I and III (GSD I and GSD III) are also characterized by mitochondrial dysfunctions. Here, Hannibal et al. provided a metabolite characterization of fibroblasts from patients with either GSD I or III and provided extra- and intracellular metabolite profiles that distinguish glycogen storage disease subtypes from healthy controls.

The effects of altered mitochondrial dynamics in the nervous system and during neurodegeneration and aging have also been widely recognized. Areas of the brain that sense changes to hormonal and nutritional status can present altered mitochondria fission and fusion events. In a comprehensive literature review, Haigh et al. highlight how changes in mitochondrial dynamics trigger modifications in neuronal signaling, and alteration in the ability of the brain to modulate metabolic functions such as feeding behavior and blood glucose levels. In the brain, the dysregulation of mitochondrial dynamics affecting energy production, such as mutations in the mitochondrial DNA that alter the assembly of the respiratory chain, have been associated with the development of neurodegenerative conditions such as Alzheimer’s and Parkinson’s disease, as presented here by Novack et al.

The secretion process associated with the release of hormones by endocrine cells, cytokines or antibodies by immune cells, and neurotransmitters from neuronal synapses require energy and calcium release. Mitochondria are key players in this process. Here, Martínez et al. investigated the role of mitochondrial dynamics and metabolism in the secretion process by different cell types in physiological and pathological settings. The authors found that mitochondria are required for the successful transport and release of proteins to the extracellular space, and that mitochondrial dynamics as well as their interactions with other organelles, in particular with the endoplasmic reticulum, undergo profound changes in response to secretion stimuli.

Several pathologies are associated with increased levels of pro-inflammatory stimuli that, in the long-term, overwhelm the local regulatory mechanism causing systemic inflammation. This is the case for obesity where increased free fatty acids (FAs) in the circulation cause low-grade inflammation that alters many endocrine pathways. Branched-chain amino acids (BCAAs) and FAs participate in mitochondrial biogenesis, energy metabolism, and inflammation. Ye et al. contributed to this Research Topic with a comprehensive revision on how increased levels of BCAA and FAs can alter mitochondria biogenesis and ATP production, which in turn, can trigger insulin resistance and activate the NFkB signaling pathway. On the other hand, chronic low-grade inflammation triggered by innate immunity has been also associated with the development of type 2 diabetes. In particular, Chang et al. used cybrid B4 cells harboring a diabetes-susceptible mitochondrial DNA haplogroup to demonstrate that the insulin sensitivity of these cells was altered by changes in mitochondria dynamics. Increased expression of fusion-related proteins led to a decrease in the inflammatory status and increased insulin sensitivity. Mitochondria are able to perceive pro-inflammatory signals and can be dysregulated during the systemic inflammatory response. Mitochondrial dysfunctions are characterized by increased production of reactive oxygen species (ROS) that triggers oxidative stress and damage to cellular organelles. External environmental factors can also contribute to the rise in systemic inflammation. Here, Magnani et al. explored how exposure to air particulate matter can stimulate inflammatory response in the body and affect the mitochondria.

Is it possible to improve the physical and metabolic status of an individual by protecting the mitochondria or by improving mitochondrial dynamics and biogenesis? This question was put to test in a model of spontaneously hypertensive rats where hypertension reduces the mitochondrial response in myocardium. Quiroga et al. showed that while in healthy rats moderate exercise improved mitochondria dynamics and biogenesis, this response was lost in hypertensive rats.

In summary, mitochondria play a fundamental role in maintaining a finely tuned energy balance in the cells; dysregulation of mitochondrial activity is associated with a wide range of diseases. Understanding how mitochondria affect endocrine functions and the molecular mechanism associated with their dynamic changes is a fundamental step towards developing more effective therapeutic treatments for many pathological conditions.

## Author Contributions

MF, BF, PM and CP edited all the manuscripts included in the Research Topic and wrote the Editorial.

## Conflict of Interest

The authors declare that the research was conducted in the absence of any commercial or financial relationships that could be construed as a potential conflict of interest.

## Publisher’s Note

All claims expressed in this article are solely those of the authors and do not necessarily represent those of their affiliated organizations, or those of the publisher, the editors and the reviewers. Any product that may be evaluated in this article, or claim that may be made by its manufacturer, is not guaranteed or endorsed by the publisher.

